# Soil quality assessment based on the TDS and MDS methods in the Qinling mountains of China

**DOI:** 10.1371/journal.pone.0335384

**Published:** 2026-08-13

**Authors:** Weige Yang, Junwei Yue, Qi Liu, Shiquan Liang, Yuanyuan Ye

**Affiliations:** 1 College of Urban, Rural Planning and Architectural Engineering, Shangluo University, Shangluo, China; 2 Shaanxi Field Research Station of Ecohydrology at the Southern Qinling Mountains, Shangluo, China; 3 Qinling Ecological Environment Research Institute, Shangluo University, Shangluo, Shaanxi, PR China; 4 College of chemical engineering and modern materials, Shangluo University, Shangluo, Shaanxi, PR China; University of Minnesota, UNITED STATES OF AMERICA

## Abstract

Artificial forests can improve the function and quality of poor soil. Different artificial trees are grown in some areas worldwide. However, data on soil quality levels in different artificial forest soils in the Qingling Mountains of China are scarce. The purpose of this research was to estimate the level and spatial distribution characteristics of soil quality by applying the total dataset (TDS) and minimum dataset (MDS) methods and to verify the accuracy of the results using the MDS methods for different artificial forestland soils in the Qingling Mountains of China. Twenty-six soil samples were collected from the *Walnut, Robinia pseudoacaci*, *Broussonetia papyrifera*, *Pinus psammophila,* and *Quercus palustris Münchh* soil and 13 soil quality indicators comprising soil physical and chemical properties were analyzed. The values of the soil quality index (SQI) of the different artificial forest soils according to the TDS and MDS methods were in the following order: *Walnut* *>* *Robinia pseudoacaci* *>* *Pinus psammophila* *>* *Broussonetia papyrifera>* and *Quercus palustris Münchh*. The pivotal indicators for characterizing soil quality in different artificial forest soils in the Qingling Mountains were soil BD, pH, soil SOM, SP, and SWC, which were screened out by principal component analysis and the norm value. The values of the SQI ranged from 0.27 to 0.77, with an average value of 0.53, when the TDS method was used, and from 0.39 to 0.87, with an average value of 0.62, when the MDS method was used. The spatial distribution of the SQI was in the form of a strip. Moreover, a 1:1 line was used to test the accuracy of the MDS method. The coefficient of certainty was 0.778, and the coefficient of relative deviation was 0.037, which indicated that the cultivated soil quality in different artificial forestland areas in the Qingling Mountains of China could be assessed using the MDS method. Hence, to achieve the sustainable development of forestry, effective soil quality improvement measures, for instance, building the best ecological benefit of the stand and preventing the decline in soil fertility and the ecological environment through reasonable tending, should be implemented instead of current traditional forest management measures.

## Introduction

Afforestation involves the planting of grass and trees and the restoration of vegetation in unsuitable farmland, which is characterized by severe soil erosion, desertification, salinization and rocky desertification and low grain yield. This project has been implemented in China and many other places and has achieved obvious results. This widespread shift in plant species composition and coverage could affect soil physical and chemical properties [1–4] and further change soil function and quality [5]. However, the effects of vegetation restoration on soil quality are unclear because of differences in planted tree types, restoration ages and so forth [6,7].

Soil quality is the ability of soil to support biological productivity, protect the environment, and promote the health of animals and plants in a specific ecosystem [8]. It can be evaluated comprehensively by soil physical, chemical and biological properties instead of individual soil properties. Soil chemical and physical properties, which can be measured by available and simple conventional methods, are widely adopted to assess soil quality [9–12].

Many soil quality evaluation methods rely on soil indicators, such as the soil quality index (SQI) [9,13], the soil management evaluation framework [14–18], the scoring function [19], and pedotransfer function analysis [20]. The SQI is easy to use and quantitatively flexible and is the most commonly adopted means for evaluating soil quality levels [21]. At present, the SQI is usually calculated by the principal component analysis (PCA) method [13,22,23], linear and multiple regression analysis [11,21], total dataset (TDS) method [24,25], and minimum dataset (MDS) method [13,23,24]. Among those methods, the TDS and MDS approaches are often selected for evaluating soil quality. In the TDS method soil quality is evaluated by taking all the indicators as equally important. The advantage of the TDS method is that all the measured indicators are analyzed and contain all the soil information, and the results are accurate and scientific. However, the disadvantage is that the calculation requires much work, and the process is complicated. The strength of the MDS method is its **efficiency and parsimony,** and its associated challenge is the **subjectivity and statistical uncertainty** inherent in the indicator selection process. Many studies have determined the critical indicators in the MDS through PCA, and the results have shown that the MDS method in combination with PCA is the most appropriate method for evaluating soil quality [8,9,12,26,27].

The Qinling Mountains are an important mountain forest ecosystem in China. The soil quality of artificial forestland plays an important role in forestry improvement and ecological environmental development. In the past, studies on the soil quality of artificial forests in the Qinling Mountains focused mainly on some properties of artificial forestland and farmland [28–32]. However, comprehensive evaluations of the soil quality of artificial forests in the Qinling Mountains based on the minimum dataset and full dataset methods is still rare. Therefore, it is necessary to assess soil quality on the basis of the integration of soil physical and chemical indicators using the TDS and MDS methods and to further test the accuracy of the MDS results. The TDS method provides a robust, full-information baseline, whereas the MDS method offers a pragmatic tool for identifying the most influential, manageable indicators for potential future monitoring. This dual approach enhances the credibility and utility of our assessment.

The objectives of this study were (1) to quantify changes and spatial characteristics in soil chemical and physical indicators in the different artificial forestland soils in the Qinling Mountains; (2) to assess soil quality using the TDS and MSD methods through PCA and investigate the spatial variation in soil quality; and (3) to test the precision of the results using the MDS method.

## Materials and methods

### Study area

**Fig 1:**
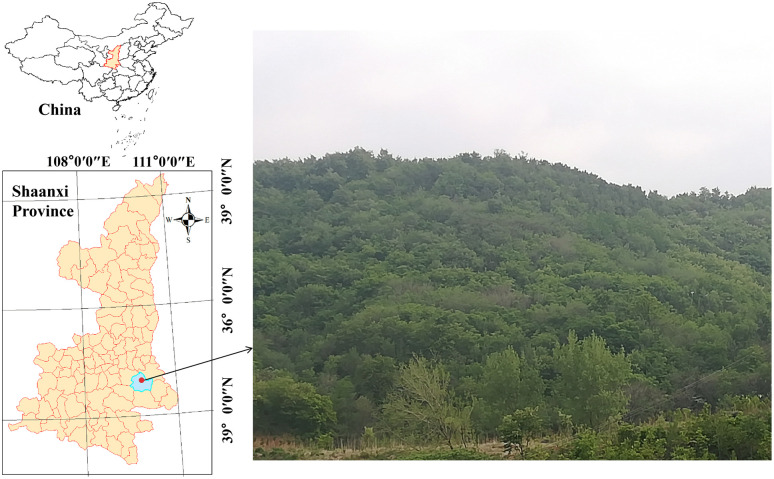
Location of Jin Feng mountain in China.

This study was conducted on Jin Feng Mountain, which is on the south slope of the Qingling Mountains and is located in the Shangzhou district (33°63′ ~ 34°18′ N, 109°50′ ~ 110°25′ E), Shangluo municipality, northern Shaanxi Province, China (Fig 1). The topographical features in this region are low mountains and hills, with higher elevations in the northwest and lower elevations in the southeast. Its landscape is typified by a structural ratio of 70% mountains, 20% cultivated land/terraces, and 10% rivers and water bodies. The study region has a warm temperate semihumid continental monsoon climate characterized by hot, rainy summers and mild, relatively dry winters. The mean annual temperature is 13.5 °C. The average annual precipitation is 758 mm, and 50% of the rain is received during the period from July to September. Luvisols (World Reference Base for Soil Resources) is the dominant soil type in the area. The dominant vegetation types include deciduous broad-leaved forests and mixed deciduous-evergreen broad-leaved forests. We focused on the most widespread plantation types in the region, which are *Walnut*, *Robinia pseudoacaci*, Chinese pine (*Pinus psammophila*), black locust (*Robinia pseudoacacia*), and secondary forests dominated by native oak species (*Quercus palustris Münchh*). These species represent the key silvicultural choices for soil and water conservation and timber production in the southern Qinling Mountains.

### Soil sampling

First, a detailed field survey, which included assessing differences in topography, soil type and plantation wood species using a 1:10 000 topographic map and a 1:10 000 land use map of the Shang Zhou district, was conducted. Jinfeng Mountain was selected as the study area. There are *Walnut, Robinia pseudoacaci*, *Broussonetia papyrifera*, *Pinus psammophila,* and *Quercus palustris Münchh* are the representative planted wood species in the Qinling Mountains. Moreover, the climate and aspect of the artificial forests are the same in the sampling area. Second, a 50-m-long × 20-m-wide grid was used to collect soil samples to capture the full range of environmental gradients on the hillslope. The soil samples were collected in March 2018. A suite of site characteristics, including plantation type, elevation, and slope position, was recorded for all the sampling plots (Table 1). Third, the Magellan global positioning system tracker, whose precision was 5 m, was used to measure the geographic coordinates and altitude of each sampling site at the same time as the sampling.

**Table 1:** Basic characteristics of the soil sampling plots.

Forest type	Attitude (m)	Slope (°)	Aspect	Age (a)
***Walnut***	760	0	Southeast	12
***Robinia pseudoacaci***	780	5	Southeast	21
***Broussonetia papyrifera***	800	40	Southeast	21
***Pinus psammophila***	830	50	Southeast	21
***Quercus palustris Münchh***	860	60	Southeast	21

Each soil sample was obtained at a 0–20 cm soil depth. We collected two of types soil samples for analysis purposes. One was the undisturbed soil, and the other was the disturbed soil. At every sampling point, an iron ring with a height of inside 5 cm and an inner diameter of 5 cm -and iron containers were used to collect the undisturbed soil for bulk density (BD) and aggregate-stability measurements, respectively. Aluminum boxes were used to collect the undisturbed soil for soil water content (SWC) measurement. Approximately 1 kg of each mixed soil sample was used to assess the soil chemical and physical properties. The disturbed soil sample at each sampling sites was an adequate composition of four subsamples, which were taken in a 1 m^2^ area. Three replicate samples were obtained for each sampling site. In total, 26 soil samples were gathered, packed in plastic bags and brought back to the laboratory within two days.

Afterward, the soil samples were air dried in the laboratory. The gravel pieces of crop roots, residues, and other coarse materials in the samples were removed manually. After that, the remaining soil was ground in a bowl and passed through two sieves of different sizes. One portion was passed through a 0.25-mm mesh to determine the soil organic matter (SOM) and total nitrogen (TN, the sum of all organic and inorganic nitrogen forms). One portion was passed through a 1-mm mesh to measure nitrate nitrogen (NH_3_^-^-N), ammonium nitrogen (NH_4_^+^-N), total phosphorus (TP), available phosphorus (AP, the phosphate fraction extractable by 0.5 M NaHCO₃, representing plant-accessible P), and pH.

### Laboratory analysis

There were three replicates for the soil chemical and physical indicator analyses. Soil BD was determined by measuring the original soil core bulk and the dry mass, which had been dried in 105 °C in an oven [33]. The soil particle fractions were measured by laser diffraction using a Mastersizer 2000 (Malvern Instruments Company, UK), following size class definitions by the USDA [34], and are expressed as the geometric mean diameter (GMD). The GMD was calculated by Equation [1]:


$$\begin{array}{c} \text{GMD=}exp(0.01\times \sum_{i=1}^{n}{\text{f}}_{i}\;ln\;{\text{m}}_{\text{i}}) \end{array}$$ (1)

where GMD is the geometric mean diameter of the soil particles (mm), *f*_*i*_ is the weight percentage of the particle size fraction (%), *m*_*i*_ is the arithmetic mean of the particle size limits (mm), and *n* is the number of particle size fractions.

Soil aggregate stability was determined by the slow wet sieving method [35] and it is expressed as the mean weight diameter (MWD). The MWD was calculated as follows:


$$\begin{array}{c} \text{MWD=}\sum_{\text{i=1}}^{\text{n}}\text{(}\overset{―}{{\text{R}}_{\text{i}}}{\text{w}}_{\text{i}}\text{)}/\sum_{\text{i=1}}^{\text{n}}{\text{w}}_{\text{i}} \end{array}$$ (2)

Where $\overset{―}{{\text{R}}_{i}}$ is the average diameter of the class i soil aggregates, and *w*_*i*_ is the percent of class i in the whole determined soil.

Wet oxidation with K_2_Cr_2_O_7_ was used to analyze SOM [33]. The semimicro-Kjeldahal method was used to measure TN [33]. The concentrations of NH_3_^-^-N and NH_4_^+^-N in the soil were measured by extraction with a 1 M KCl solution [33]. Soil TP and AP were measured by extracting samples with 0.5 M NaHCO_3_ and assaying P calorimetrically with molybdate [33]. Soil pH was determined by an automatic acid‒base titrator in 1:2.5 soil-to-water suspensions [33].

### Soil quality assessment

The data analysis steps of in the TDS method were as follows: (1) obtain the common factor variance matrix and calculate the weight value by factor analysis for all the indicators; (2) calculate the membership degree of all the indicators; and (3) compute the comprehensive SQI of each soil sample according to the membership degree and weight. The steps in the MDS method were as follows: (1) carry out correlation analysis and KMO value tests for all indicators; (2) determine the minimum dataset index system using PCA; and (3) calculate the SQI.

There were three steps to compute the SQI using PCA: (1) singling out the key indicators; (2) scoring the indicators according to their soil function performances in converting the indicator values into dimensionless scores ranging from 0 to 1; and (3) integrating the indicator scores into a soil quality index. On the basis of the indicator correlations, PCA was executed to divide the standardized data properties into principal components (PCs). To maximize the correlations between the PCs and soil properties, the factors needed to be subordinate to varimax rotation. Every variate was correlated with the attribute group that included it. Eigenvalues > 1 were retained to interpret the data variation. For every PC, the highly weighted indicators were the soil properties for which the absolute values of the weight load were within 90% of the highest weighted loading. If one or more variables were kept in the PC and were not correlated with each other (*r <* 0.60), they were regarded as critical indicators and were retained in the MDS [11]. Furthermore, the indices selected for the MDS must be uncorrelated with each other. Correlation analysis was carried out for each group. If *r* < 0.5, all the indexes were selected for the MDS. If *r* > 0.5, the indicator for which the norm value was the greatest was selected for the MDS.

The norm value is extensively used to select the appropriate MDS indicators. The norm value represents the comprehensive load of the soil indicators. The larger the norm value is, the more the soil quality information can be represented. Its calculation formula is as follows [36,37]:


$$\begin{array}{c} \begin{array}{c} {\text{N}}_{\text{ik}}=\sqrt{\sum_{\text{i}}^{\text{k}}{\text{(U}}_{\text{ik}}^{\text{2}}\lambda_{k}\text{)}} \end{array} \end{array}$$ (3)

Where *N*_*ik*_ is the norm; *U*_*ik*_ is the load of the *i*th variable in the principal component; and *k* is the eigenvalue of the principal component [36,37].

To eliminate the influence of different measurement units on the computations, the overall data should be standardized prior to the PCA analysis as follows:


$$\begin{array}{c} \text{Type S}:\;f(x)=\left\{ \begin{array}{c} @c\text{0}.\text{1}\\ \text{0}.\text{9}\times (x-a)/(b-a)+\text{0}.\text{1}\\ \text{1} \end{array}\right.\;\begin{array}{c} x\leq a\\ a<x<b\\ x\geq b \end{array} \end{array}$$ (4)

$$\begin{array}{c} \text{Type reverse S}:\;f(x)\;\left\{ \begin{array}{c} @c\text{0}.\text{1}\\ \text{0}.\text{9}\times (b-x)/(b-a)+\text{0}.\text{1}\\ \text{1} \end{array}\right.\;\begin{array}{c} x\leq a\\ a<x<b\\ x\geq b \end{array} \end{array}$$ (5)

*f(x)* is the indicator scoree, which varies from 0.10 to 1.00; *x* is the indicator value; *a* represents the minimum value of the indicator and *b* represents the maximum value of the indicator. Only the soil BD was standardized calculated by Equation [5], and the other soil indicators were standardized by Equation [4].

A reasonable scientific soil quality evaluation method should consider not only soil property interactions but also the influence of every soil property weight on soil quality evaluation. A weighted method was employed as follows on the basis of the scientific literature [38]:


$$\begin{array}{c} SQI=\sum_{i=1}^{n}W_{i}S_{i} \end{array}$$ (6)

The SQI is the soil quality index, which varies from 0 to 1. A higher SQI indicates means better soil quality. Here, *W*_*i*_ is the weighting factor of the PC, *S*_*i*_ is the indicator score of each variable *i*, and *n* is the number of variables in the MDS. The values of *W*_*i*_ and *S*_*i*_ must be determined prior to applying this equation. After the key indicators in the MDS were confirmed, the weight of every indicator in the MDS was computed. The weight of the indicator was the quotient of its communality and the sum of all the indicator communalities in the MDS [39]. In accordance with the SQI values of forestland, the SQI was divided into five grades: lowest (0 ≤ SQI ≤ 0.2), lower (0.2 < SQI ≤ 0.4), medium (0.4 < SQI ≤ 0.6), high (0.6 < SQI ≤ 0.8), and highest (0.8 < SQI ≤ 1).

### Data analyses

The data were analyzed using traditional statistical approaches, including Pearson correlation coefficients and PCA, in SPSS 16.0 software. Pearson correlation analysis was applied to identify the relationships among the indicators and the soil quality index (SQI). This method was chosen because of its appropriateness for continuous data and its prevalence in soil science, facilitating comparison with prior studies. Prior to analysis, the normality of the variables was assessed using the Shapiro‒Wilk test. Variables that deviated significantly from normality were log-transformed to better meet the assumption of bivariate normality. The robustness of the linear correlation results was further verified by comparing them with nonparametric Spearman rank correlations, which yielded consistent patterns. Spatial distribution maps of the soil properties and the SQI were constructed using Surfer 8.0 software. A 1:1 linear relationship between SQI_MDS_ and SQI_TDS_ was adopted to test the accuracy of SQI_MDS_ using SigmaPlot 12.5 software. All the tests in this study were performed at the 0.05 or 0.01 significance level. Afterward, the coefficient of certainty (EF) and the coefficient of relative deviation (ER) were applied to evaluate the prediction precision of the simulation values [40]. The EF value represents the degree of relationship between the observed values and the estimated values. The ER value represents how well the calculated value is compared with the observed value. If the EF value is close to 1 and the ER is close to 0, the simulated value is close to the measured value. If the EF value is close to 0 and ER value is close to 1, the simulated value is considered‘poor’.

## Results and discussion

### Soil physical and chemical indicators

The 26 sampling points and the measured 13 soil chemical and physical properties made up the data matrix. Dispersion, including the range, mean and standard deviation (SD), and coefficient of variation (CV), among the 13 soil properties was calculated by applying classical statistical methods (Table 2).

**Table 2:** Degree of dispersion in soil quality indicators.

Soil indicators	Min	Max	Mean	Median	SD†	CV, %‡
Soil bulk density (BD), g cm^–3^	0.93	1.50	1.15	1.14	0.12	10.79
Soil water content (SWC), %	10.36	20.84	14.93	14.62	3.16	21.14
Saturated moisture (SM), %	28.96	69.80	50.44	50.17	9.16	18.16
Soil porosity (SP), %	43.42	75.80	66.18	64.75	6.54	9.89
Mean weight diameter (MWD), mm	1.35	2.96	2.23	2.21	0.50	22.31
Geometric mean diameter (GMD), mm	0.73	2.56	1.62	1.54	0.58	36.06
pH	5.73	7.39	6.40	6.10	0.50	7.88
Soil organic matter (SOM), g kg^–1^	5.26	33.18	25.17	26.13	6.06	24.09
Total N (TN), g kg^–1^	1.07	5.50	2.77	2.66	1.13	40.72
NH_3_^-^-N, mg kg^–1^	0.21	4.18	1.40	1.26	1.03	73.61
NH_4_ ^+^ -N, mg kg^–1^	10.91	140.05	63.07	42.69	46.76	74.14
Total P (TP), g kg^–1^	0.53	5.66	1.52	1.02	1.18	78.03
Available P (AP), mg kg^–1^	1.33	10.25	4.39	3.34	2.68	61.10

† SD: standard deviation; ‡ CV: coefficient of variation.

Different soil indicators at the 26 sampling sites displayed different degrees of heterogeneity (Table 2). The SOM concentrations in the artificial forestland varied from 5.26 to 33.18 g kg^–1^, with a mean of 25.17 g kg^–1^. On the basis of the soil nutrient classification criteria of the Second National Soil Survey in China [41], the SOM level was considered medium. This finding is consistent with results of Qi Lu’s study [42]. On the one hand, the topography in the study area is complex, and artificial trees grow on high-elevation hills and mountains where the soil is thinner. On the other hand, these four artificial trees are in deciduous broad-leaved forests, the amount of litter under the forest is greater, the litter is easier to decompose, and the organic matter in the soil increased.

The average TN content was 2.77 g kg^–1^, which is at the highest level according to the soil nutrient classification criteria [41]. The TN concentration ranged from 1.07 to 5.50 g kg^–1^. The average the TP content was 1.52 g kg^–1^, which is at the highest level according to the soil nutrient classification criteria [41]. The TP concentration ranged from 0.35 to 5.66 g kg^–1^. Two reasons could explain for the higher TN and TP contents in the soil. First, there is a sufficient water source and appropriate temperature for nitrogen and phosphorus accumulation in this area. In addition, many the broad-leaved tree species contain litter that decomposes faster and more easily, leading to high soil nutrient levels [43,44].

The mean AP content was 4.39 mg kg^–1^ (Table 1), which is at the low level on the basis of published soil nutrient classification criteria [41]. The average NH_3_^-^-N and NH_4_^+^-N contents were 1.40 and 63.07 mg kg^–1^, respectively. The soil pH of the sampled cultivated land ranged from 5.73 to 7.39, with an average value of 6.40, which indicated that the soils are slightly acidic. The BD ranged from 0.93 to 1.50 g cm^–3^, with an average value of 1.15 g cm^–3^, which is considered suitable for soil according to the soil nutrient classification criteria of the China Second National Soil Survey [41].

The coefficient of variation is used to judge the stability of the data distribution in space. The greater the CV value is, the worse the stability and the greater the interference from human factors [42]. In this study, on the basis of the classification by Hillel [45], soil pH and SP demonstrated weak spatial variability (CV < 10%), some tested soil indicators demonstrated moderate variability (10% < CV < 100%), in the order of BD < SM < SWC < MWD < SOM < GMD < TN < AP < NH_3_^-^-N < NH_4_^+^-N, and no tested soil indicators had strong variations (CV > 100%) (Table 2). The CVs of the soil physical indicators (19.73%) were lower than those of the soil chemical indicators (51.37%) (Table 2). Soil BD and SP were stable and uniform and rarely affected by anthropogenic or external effects. Soil carbon, nitrogen and phosphorus showed moderate spatial variability because they were easily affected by anthropogenic factors, such as the degree and mode of management.

Several soil chemical–chemical, physical–physical, and chemical–physical parameters were strongly correlated (Table 3). The greatest significant positive correlations were obtained between the MWD and GMD (*r* = 0.993, *P* < 0.001), TN and GMD (*r* = 0.516, *P* < 0.001), NH_4_^+^-N and MWD (*r* = 0.712, *P* < 0.001), NH_4_^+^-N and GWD (*r* = 0.729, *P* < 0.001), NH_4_^+^-N and TN (*r* = 0.582, *P* < 0.001), AP and MWD (*r* = 0.559, *P* < 0.001), AP and GWD (*r* = 0.581, *P* < 0.001), AP and TN (*r* = 0.573, *P* < 0.001), and AP and NH_4_^+^-N (*r* = 0.753, *P* < 0.001). In addition, significant negative correlations were detected between SM and BD (*r* = −0.967, *P* < 0.001), and between NH_3_^-^-N and pH (*r* = −0.600, *P* < 0.001) (Table 3). There were 91 pairs of correlations among the soil indicators, as shown in Table 2. Seventeen pairs were significantly correlated (*p <* 0.05). This frequency of the correlations between soil properties demonstrated that the determined soil properties could be classified into several categories according to their correlation modes.

**Table 3:** Correlation (r) between measured indicators in the studied catchment.

Soil properties	BD	SWC	MWD	GWD	SM	SP	pH	SOM	TN	NH_3_^-^-N	NH_4_ ^+^ -N	TP	AP
**BD**	1												
**SWC**	0.015	1											
**MWD**	–0.174	0.190	1										
**GWD**	−0.118	0.238	0.993**	1									
**SM**	−0.967**	−0.003	0.230	0.180	1								
**SP**	–0.224	0.110	0.163	0.138	0.191	1							
**pH**	0.016	−0.039	−0.155	−0.130	0.027	−0.287	1						
**SOM**	–0.023	−0.105	0.430*	0.409*	0.113	−0.226	0.219	1					
**TN**	0.065	0.454*	0.479*	0.516**	−0.072	0.246	−0.322	−0.300	1				
**NH**_**3**_^**-**^ **-N**	–0.276	0.257	0.189	0.184	0.250	0.034	−0.600**	−0.277	0.450*	1			
**NH**_**4**_ ^+^ **-N**	–0.075	0.251	0.712**	0.729**	0.143	−0.135	−0.288	0.318	0.582**	0.298	1		
**TP**	0.228	−0.019	0.148	0.161	−0.160	−0.029	−0.154	0.157	0.180	0.114	0.265	1	
**AP**	–0.191	0.352	0.559**	0.581**	0.202	0.021	−0.145	0.262	0.573**	0.417*	0.753**	0.163	1

* indicates a significance level at *P* < 0.05; ** indicates a significance level at *P* < 0.01.

### Spatial characteristic of soil physical and chemical indicators

**Fig 2:**
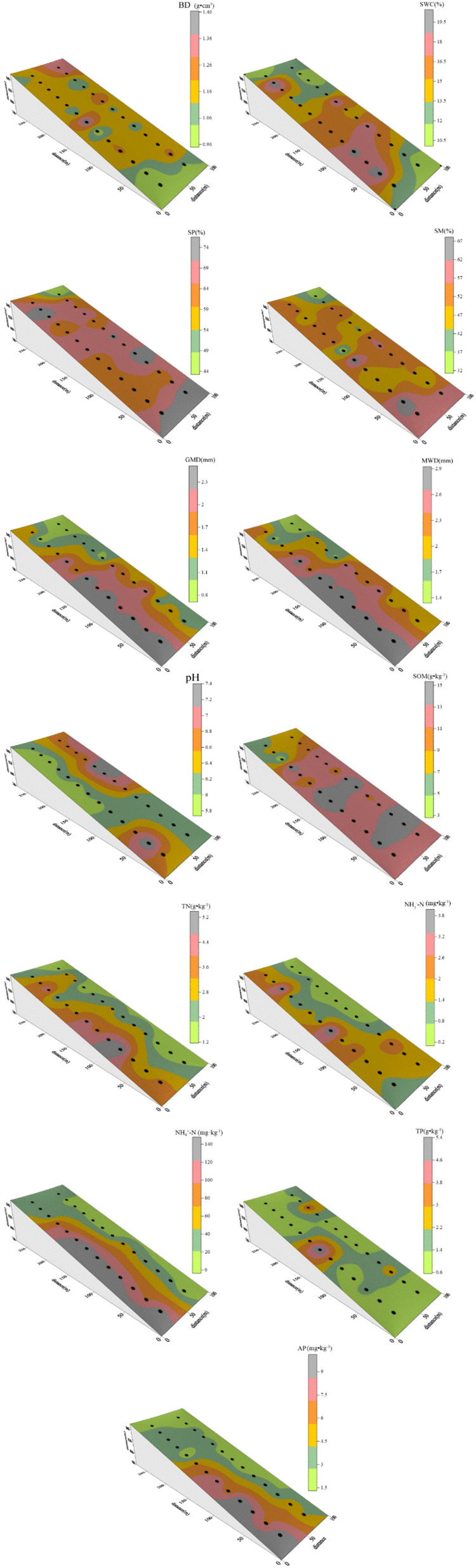
Spatial distribution of soil physical and chemical properties.

We used the spatial interpolation procedure to estimate soil physical and chemical indicators in the study catchment at unsampled locations from the available data. The results of this spatial interpolation are shown in Fig 2. The spatial distribution of BD showed nonlinear variation along the slope. The soil BD increased from the lower slope are to the mid-slope, where the maximum BD (1.50 g·cm ⁻ ³) occurred. Afterward, it decreases toward the upper slope. The spatial distribution of BD was complex and may have been influenced by the combined effects of topography, erosion, and moisture redistribution along the slope. The spatial distributions of SWC and SP were mainly banded, supplemented by patchy distribution at some sampling points. The SWC first increased but then decreased from the lower slope to the upper slope, whereas the SP decreased from the lower slope to the upper slope. The spatial distributions of the MWD, GWD, and AP content exhibited a banded pattern, which decreased from the lower left to the upper right on the slope. The soil pH also exhibited in a banded pattern and first decreased but then increased from the lower left to the top right of the slope. The spatial distribution of SOM was complex and irregular along the slope. The maximum SOM contents were located in the middle and lower parts of the slope, and the minimum SOM contents were located at the top of the slope. There were some local patches with different SOM contents, indicating that factors other than the overall slope gradient, such as local differences in soil erosion, water retention, and vegetation cover, may also affect the distribution of SOM. Some circular or oval – shaped areas with relatively high or low SOM contents were observed, suggesting presence of local microenvironmental factors that influence the accumulation or loss of SOM. The spatial distributions of the TN, NH_3_^-^-N and NH_4_^+^-N contents exhibited a banded pattern and displayed a decreasing trend from the left to the right of the slope. The spatial distribution of the TP contents was patchy and striped, and the TP content decreased from the middle slope to the upper and lower slope.

### Soil quality evaluation using the TDS method

**Fig 3:**
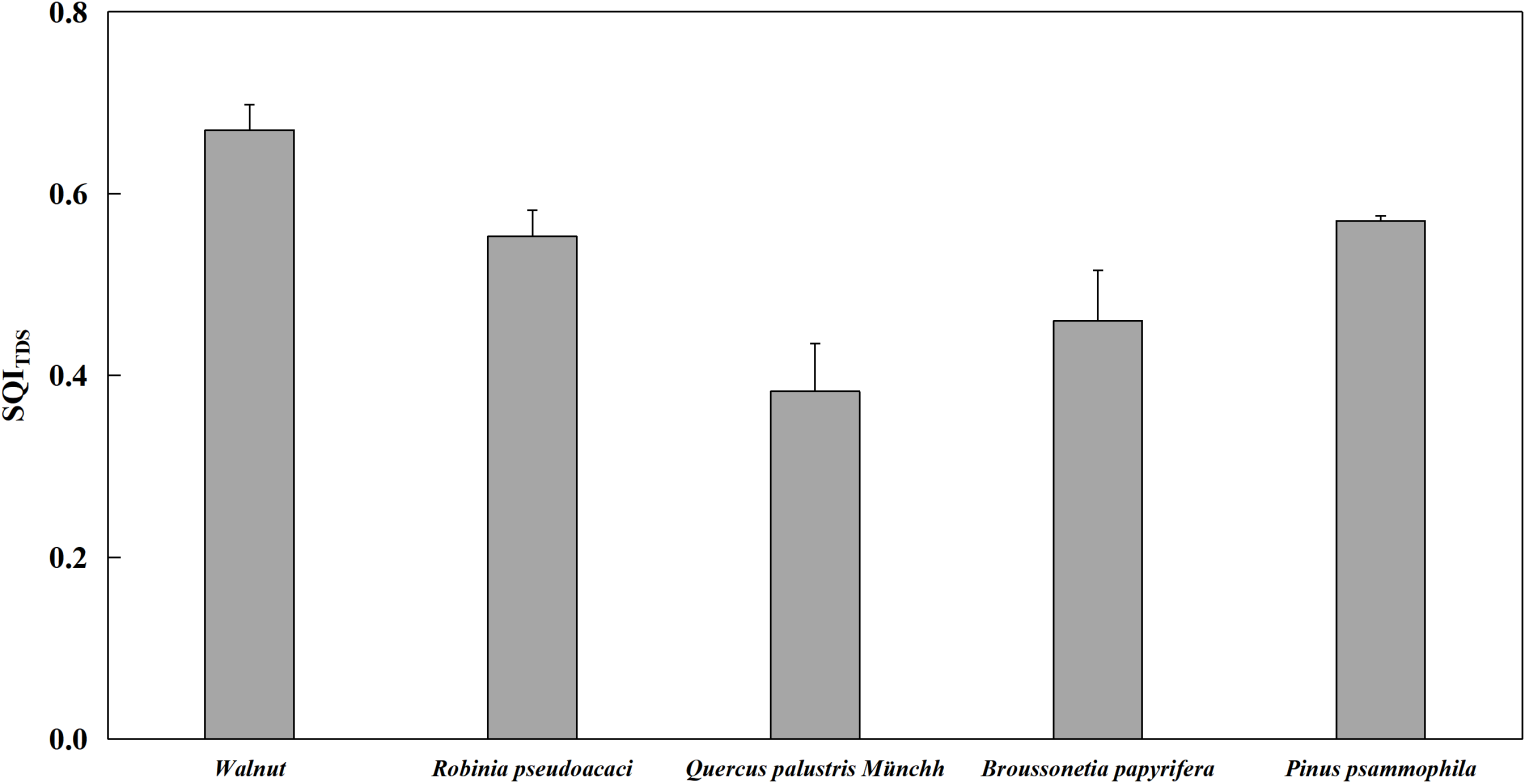
SQI_TDS_ values of different artificial forest soils.

The results indicated that the SQIs of the 26 sampling sites obtained using the TDS method changed from 0.26 to 0.77, and the average value was 0.53, and the variation coefficient was 24.14%. Moreover, the SQIs of the 7 sampling sites were greater than 0.6: 16 sampling sites ranged from 0.6 to 0.4, and 3 sampling sites ranged from 0.2 to 0.4. There were no sampling sites whose indices were greater than 0.8. The above mentioned data indicate that the soil quality level is moderate. The SQI_TDS_ values of 62% of the sampling sites were at the medium level, whereas 27% of the sites were at the high level. The average SQI_TDS_ values of different artificial forest soils were in the order of *Walnut* *>* *Robinia pseudoacacia >* *Pinus psammophila* *>* *Broussonetia papyrifera* *>* *Quercus palustris Münchh* (Fig 3). The SQI_TDS_ values in *Walnut* forest soil ranged from 0.58 to 0.73, with a mean of 0.67. The soil quality level of the *Walnut* forest soil was relatively high. The SQI_TDS_ of the *Robinia pseudoacaci* forest soil ranged from 0.44 to 0.77, with an average of 0.58, which indicated that soil quality of the *Robinia pseudoacaci* forest soil was at a medium level. The SQI_TDS_ values in the *Broussonetia papyrifera* forest soil ranged from 0.39 to 0.57, and the mean was 0.46. The soil quality level of the *Broussonetia papyrifera* forest soil was moderate. The SQI_TDS_ values in the *Quercus palustris* Münchh forest soil ranged from 0.26 to 0.48, with a mean of 0.38 (Fig 3).

The soil quality level of *Quercus palustris Münchh* forest soil was low. The main reason for the higher soil quality of *Walnut* and *Robinia pseudoacaci* was that the soil sedimentation occurred on the lower slope. Therefore, the soil layer was thicker, and the soil nutrients were enriched in the *Walnut* and *Robinia pseudoacaci* forest soil. In contrast, the steep slope and soil erosion in *Quercus palustris Münchh* forest soil led to soil thinning, nutrient loss, and low soil quality. Obviously, identifying the limiting factors of *Quercus palustris Münchh* forest soil quality and implementing the soil quality control measures to improve the level of *Quercus palustris Münchh* forest soil quality were necessary. It is evident that the soil quality level is closely related to slope position. This pattern of enrichment at lower slopes and depletion at ridge tops is a typical manifestation of spatial heterogeneity in soil properties within mountainous ecosystems. Studies have indicated that broadleaf forests are more effective at maintaining soil fertility and promoting soil nutrient functions [46]. However, in this study, the mean soil quality of *Broussonetia papyrifera* was lower than that of *Pinus psammophila*. This may be attributed to the shallow root system and strong horizontal expansion of *Broussonetia papyrifera*, along with its fine root secretions that are rich in oxalic acid and citric acid. Concurrently, the decomposition of litter in coniferous forests generates relatively high levels of organic acids [47], which can activate aluminum- and iron oxide-bound phosphorus in the soil, leading to the accumulation of soil nutrients. Studies have shown that similar site conditions and different forests lead to variations in soil nutrients and tree growth [48]. Although the natural conditions of the five forests in the study area are similar, the driving factors of soil quality (such as nutrients, microtopography, and slope position) differ. Direct or indirect interactions among these factors may influence soil quality.

**Fig 4:**
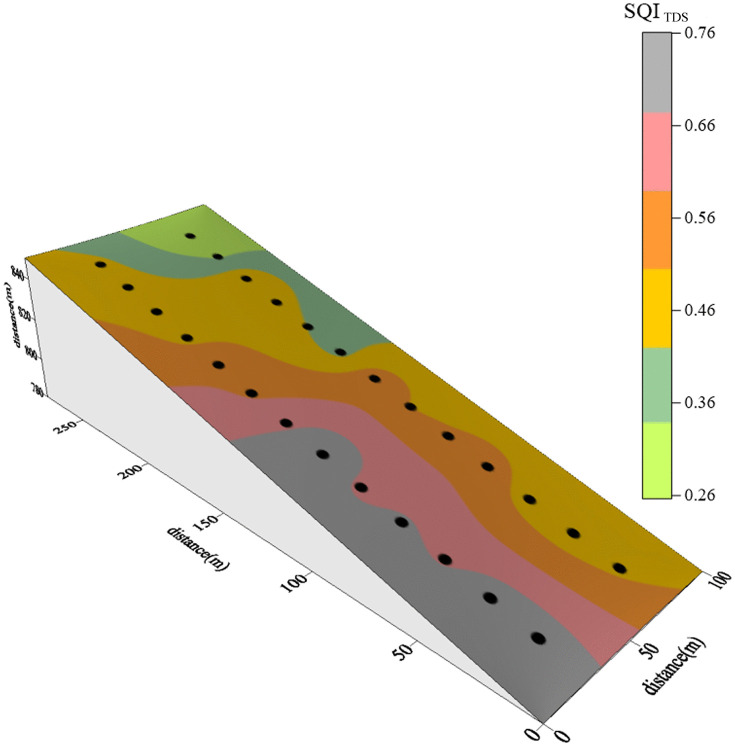
Spatial distribution of the SQI_TDS_ values.

Furthermore, the spatial distribution of the SQI_TDS_ values on the slope was striped. The SQI_TDS_ values on the slope tended to decrease from the bottom left to the top right. The maximum value occurred on the lower slope, and the minimum value occurred on the upper slope. In addition, the soil quality level on the slope was moderate, which was reflected in the area with soil quality values between 0.4 and 0.6, which occupied the largest area on the slope, and the areas with soil quality values greater than 0.6 and less than 0.4 were small (Fig 4).

### Soil quality evaluation using the MDS method

Compared with the MDS method, the TDS method markably increases the cost of laboratory analysis and the amount of labor. The SQI calculated using the TDS method is more complicated than that calculated using the MDS method [49]. Hence, the establishment of the MDS method, which is appropriate for soil quality evaluation, is widely approved. The few soil indicators carefully chosen in the MDS method provide sufficient information for soil quality evaluation [5,49,50].

To identify the pivotal soil quality properties that are regarded as the key soil quality indicators for the MDS method, all the dataset were subjected to PCA. In general, varimax rotated PCA was adopted because of the logical grouping of the variables under different factors and their higher correlation frequency between some properties. The eigenvalues determined by PCA revealed five principal components, each of which had an eigenvalue > 1 and made up 83.28% of the total variance. The percentages of variance explained by PC1, PC2, PC3, PC4, and PC5 were 32.75%, 16.45%, 15.94%, 8.88%, and 8.30%, respectively (Table 4).

**Table 4:** Results of the principal component analysis of soil quality indicators.

Soil properties	PC1†	PC2†	PC3†	PC4†	PC5†	Norm
**pH**	−0.368	0.042	0.595‡	0.478	−0.231	0.67
**NH**_**3**_^**-**^ **-N**	0.862‡	0.212	0.149	−0.120	−0.156	0.49
**NH**_**4**_ ^+^ **-N**	0.516‡	−0.249	−0.550‡	−0.355	−0.306	0.66
**AP**	0.817‡	0.048	0.061	0.035	−0.267	0.48
**TP**	0.242	0.424	−0.027	−0.509‡	0.165	0.59
**TN**	0.717‡	0.198	−0.471	0.246	−0.007	0.55
**MWD**	0.852‡	0.069	0.321	0.086	0.265	0.54
**GMD**	0.860‡	0.126	0.301	0.127	0.229	0.54
**SWC**	0.408	0.080	−0.293	0.571	−0.324	0.68
**SP**	0.171	−0.341	−0.337	0.277	0.753‡	0.84
**BD**	−0.258	0.926‡	−0.172	0.068	0.077	0.66
**SM**	0.300	−0.883‡	0.255	−0.084	−0.075	0.65
**SOM**	0.235	0.171	0.824‡	−0.162	0.056	0.62
**Statistical parameter**						
**Eigenvalue**	4.258	2.139	2.072	1.154	1.079	
**Contribution rate (%)**	32.753	16.454	15.941	8.88	8.302	
**Accumulative contribution rate (%)**	32.753	49.207	65.148	74.027	83.279	

† PC = principal component; ‡ underlined factor loading is considered highly weighted when it is within10% of the variation in the absolute values of the highest factor loading in each PC.

The indicators for which the absolute value of each principal component load value was greater than 0.5 were selected for the MDS method. In PC1, the indicators with load values greater than 0.5 were NH_3_^-^-N, NH_4_^+^-N, AP, TN, MWD, and GWD. Similarly, the second group included BD and SM. The third group included the pH and SOM. Similarly, the fourth group was TP, and the fifth group was SP. If the principal component factor load of the index was less than 0.3, it was divided into another group. There were five groups because there was no index with a factor load less than 0.3.

In the first group, there were no correlation coefficients less than 0.5 between the indicators and NH_4_^+^-N had the highest norm value of the group. Therefore, only NH_4_^+^-N with the highest norm value was selected as the high-weight indicator in the first group. BD and SM were in the second group, and the correlation between the two indices was greater than 0.3; BD had the highest norm value, while SM had the highest weighted index. Therefore, the two indices in the second group were set as high-weighted indices. PH and SOM were in the third group, and the correlation between the two indices in the third group was less than 0.3; thus, they were both high-weight indices in the group. TP and SWC were in the fourth group and the correlation between the two indices in the fourth group was greater than 0.5; only the SWC with the highest norm value was denoted as the high-weight index. AP was in the fifth group alone. Further correlation analysis was carried out according to the selected seven high-weight indicators (Table 5).

**Table 5:** The correlation coefficient of the index in each group.

First group					
Soil properties	NH_3_^-^-N	NH_4_ ^+^ -N	AP	TN	MWD
**NH**_**4**_ ^+^ **-N**	0.298				
**AP**	0.753^**^	0.417^*^			
**TN**	0.582^*^	0.450^*^	0.573^*^		
**MWD**	0.712^**^	0.189	0.559^*^	0.479^*^	
**GMD**	0.729_**_	0.184	0.581^*^	0.516^*^	0.993^**^
**Second group**		**Third group**		**Forth group**	
**Soil properties**	**BD**	**Soil properties**	**pH**	**Soil properties**	TP
**SM**	0.967^**^	SOM	0.219	SWC	0.517^*^

* indicates a significance level at *P* < 0.05; ** indicates a significance level at *P* < 0.01.NH_3_^-^-N.

The indicators with high weights and correlation coefficients greater than 0.5 and high norm values were selected for the final MDS. There was no index in the first group that was entered into the MDS. The BD in the second group was included in the MDS. The pH and SOM in the third group were used in the MDS. The SWC in the fourth group was used in the MDS, and SP in the fifth group was used in the MDS (Table 5 and Table 6). Finally, the final properties chosen through PCA were BD, pH, SOM, SP and SWC (Table 6). The correlation coefficient between each indicator of the MDS was lower than 0.5, and there was no linear correlation between the two indicators. There was also no overlap in the information reflected by the five indicators, which were significantly correlated with the other eliminated indexes. To a certain extent, the five indicators could replace the soil quality information of the other eliminated indices. Therefore, these five properties could be used to estimate the soil quality in artificial forest soil. Once the key simplified evaluation indicators of soil quality assessment were confirmed, there was no need to spend time and money on testing a range of other properties to assess soil quality.

**Table 6:** Correlation coefficient of the high-weight indices.

Soil properties	NH_4_ ^+^ -N	BD	SM	pH	SOM	SWC	SP
**NH**_**4**_ ^+^ **-N**	1						
**BD**	0.276	1					
**SM**	0.250	0.967^**^	1				
**pH**	−0.600^**^	−0.016	0.027	1			
**SOM**	−0.277	0.023	0.113	0.219	1		
**SWC**	0.257	−0.015	−0.003	−0.039	−0.015	1	
**SP**	0.034	0.224	0.191	−0.287	−0.226	0.110	1

* indicates a significance level at *P* < 0.05; ** indicates a significance level at *P* < 0.01.

The critical indicators in the MDS were determined using the PCA method, which is considered a suitable approach for soil quality assessment [5, 48-51]. Generally, the SOM content is positively correlated with the level of soil fertility. SOM is the most frequent indicator used in the MDS when applied for soil assessment [14,21,48,52–54]. Liu [55] and Zheng [56] reported that some soils are deficient in AP in China. Brady and Weil [57] proposed that the AP content could be used for soil fertility assessment and could be considered an important factor because of its weak mobility. BD can not only quantitatively characterize the ecological functions of soil, such as its water holding capacity, infiltration ability, erosion resistance and air permeability but also be among the important indices for measuring the quality of the soil environment. The soil BD and soil pH have been used in previous SQIs created using the MDS approach [10,51,58,59]. The SWC is an indispensable medium for the chemical, biological and physical processes of soil and a medium for the exchange of various substances between soil, plants and the environment. The SWC is a major source of water absorption by plants. It plays an important roles in the yield and quality of plants by affecting soil fertility, soil temperature and ventilation. In contrast to these previous studies, in this study, the BD, pH, SOM, SP and SWC were chosen to assess soil quality in the MDS, which included the most commonly used indicators (SOM and SP), important soil physical indicators (BD and SWC) and soil acidity and alkalinity properties (pH). Therefore, the selected pivotal indicators of soil quality evaluation are reasonable in this study.

**Fig 5:**
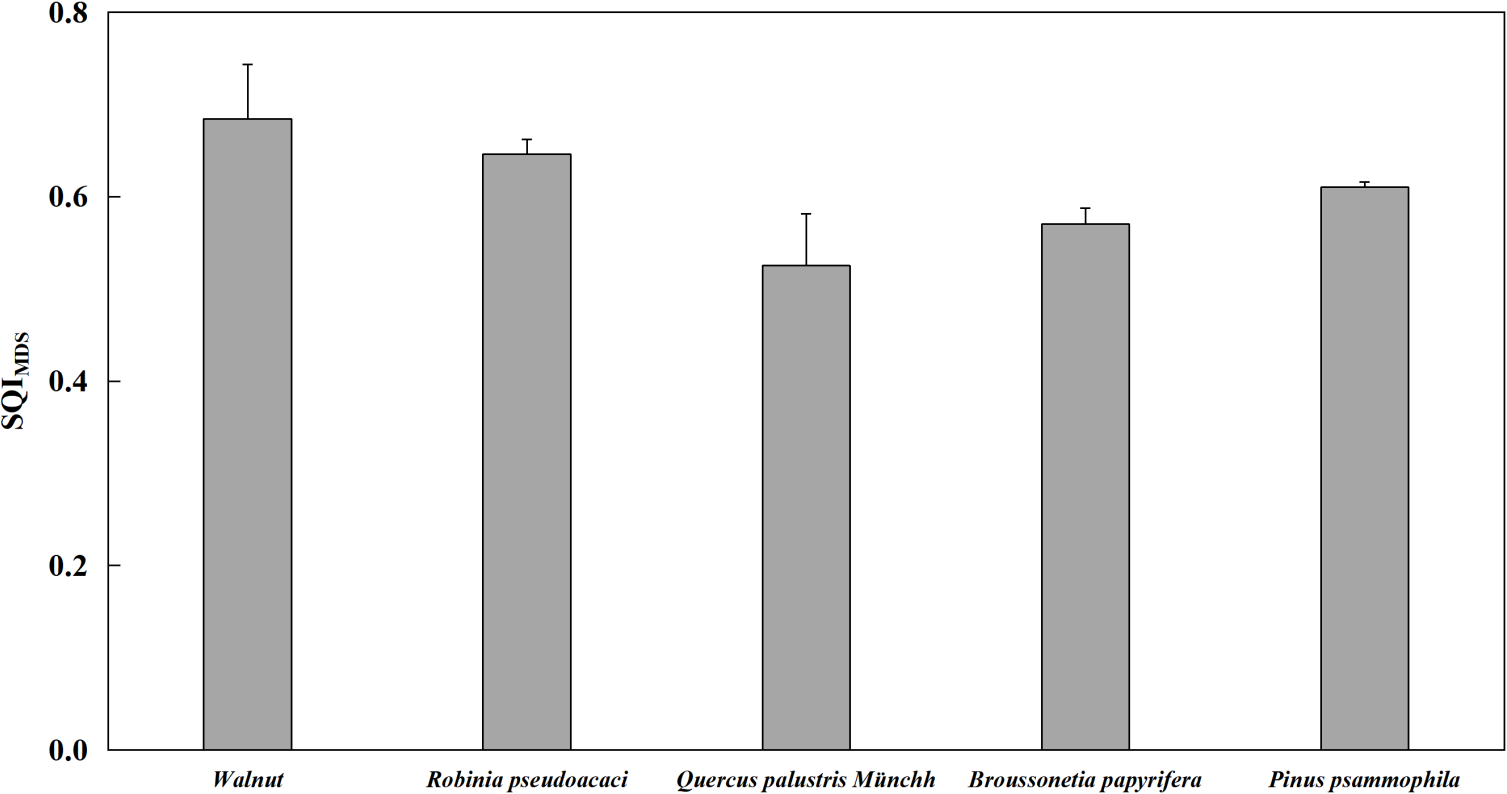
SQI_MDS_ values of different artificial forest soils.

The SQI assessment was completed in three steps. First, the BD, pH, SOM, SP and SWC indicators in the MDS were included in another PCA; Table 7 shows the communalities and weights of these five indicators. Second, the BD, pH, SOM, SP and SWC scores were calculated using Equations [4] and [6]. Finally, the SQI was computed using Equation [6]. The average value of SQIs for the 26 sampling points was 0.62, with values varying from 0.39 to 0.87 and a coefficient of variation of 13.00%. Moreover, the SQIs of the 18 sampling sites were greater than 0.6, 7 sampling sites ranged from 0.6 to 0.4 and 1 sampling site ranged from 0.2 to 0.4. Only 1 sampling site had an SQI greater than 0.8. The average SQI_MDS_ indicated that the soil quality in the study area was higher. The SQI_MDS_ values of 69% of the sampling sites were at the highest level, and 27% were at the middle level. The average values SQI_MDS_ values of different artificial forest soils were in the order of *Walnut* *>* *Robinia pseudoacaci* *>* *Pinus psammophila* *>* *Broussonetia papyrifera* *>* *Quercus palustris Münchh* (Fig 5). The SQI_MDS_ values in W*alnut* forest soil ranged from 0.53 to 0.87, with a mean of 0.68. The soil quality level of the walnut forest soil was relatively high. The SQI_MDS_ values of the *Robinia pseudoacaci* forest soil ranged from 0.53 to 0.71 with an average of 0.65, which indicated that the soil quality of the *Robinia pseudoacaci* forest soil was high. The SQI_MDS_ values in the *Broussonetia papyrifera* forest soil ranged from 0.54 to 0.60, and the mean was 0.57. The soil quality level of the *Broussonetia papyrifera* forest soil was moderate. The SQI_MDS_ values in the *Quercus palustris Münchh* forest soil ranged from 0.39 to 0.66, with a mean of 0.53. The soil quality level of *Quercus palustris Münchh forest soil* was moderate (Fig 5).

**Table 7:** Communality and weight of soil quality indicators in the MDS.

Soil indicators	Communality	Weight
**BD**	0.80	0.30
**pH**	0.45	0.17
**SOM**	0.52	0.20
**SWC**	0.23	0.09
**SP**	0.64	0.24

**Fig 6:**
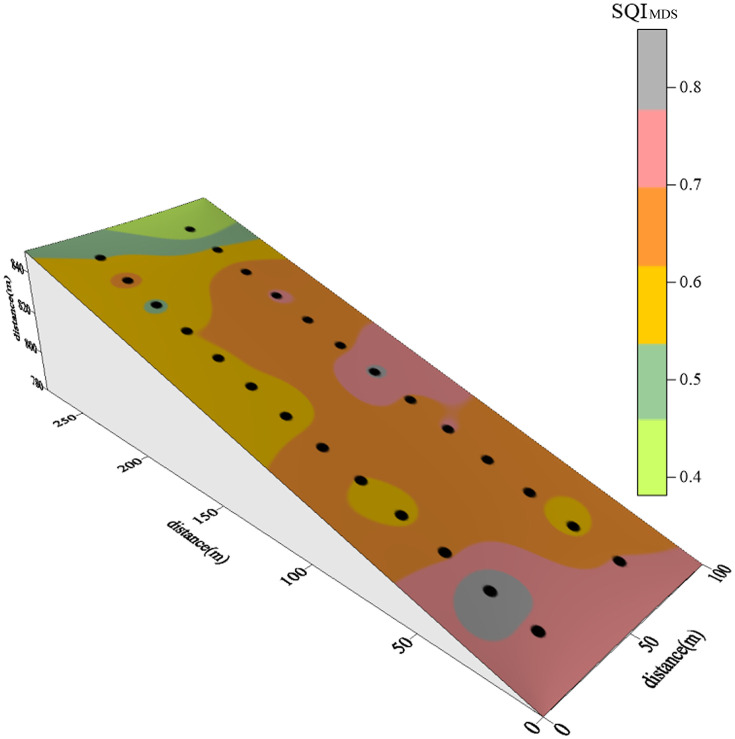
Spatial distribution of the SQI_MDS_ values.

Moreover, the spatial distribution of the SQI_MDS_ values on the slope was patchy and striped. The SQI_MDS_ values on the slope tended to decrease from the lower left to the upper right, which is consistent with the spatial distribution characteristics of the SQI_MDS_ values. The maximum value occurred on the lower slope, and the minimum value occurred on the upper slope. In addition, the soil quality level of the slope was higher, which was reflected in the fact that the area with soil quality values between 0.6 and 0.8 occupied the largest area of the slope, and the areas with soil quality values greater than 0.8 and less than 0.4 were small (Fig 6).

### Accuracy test of the results for the minimum dataset

**Fig 7:**
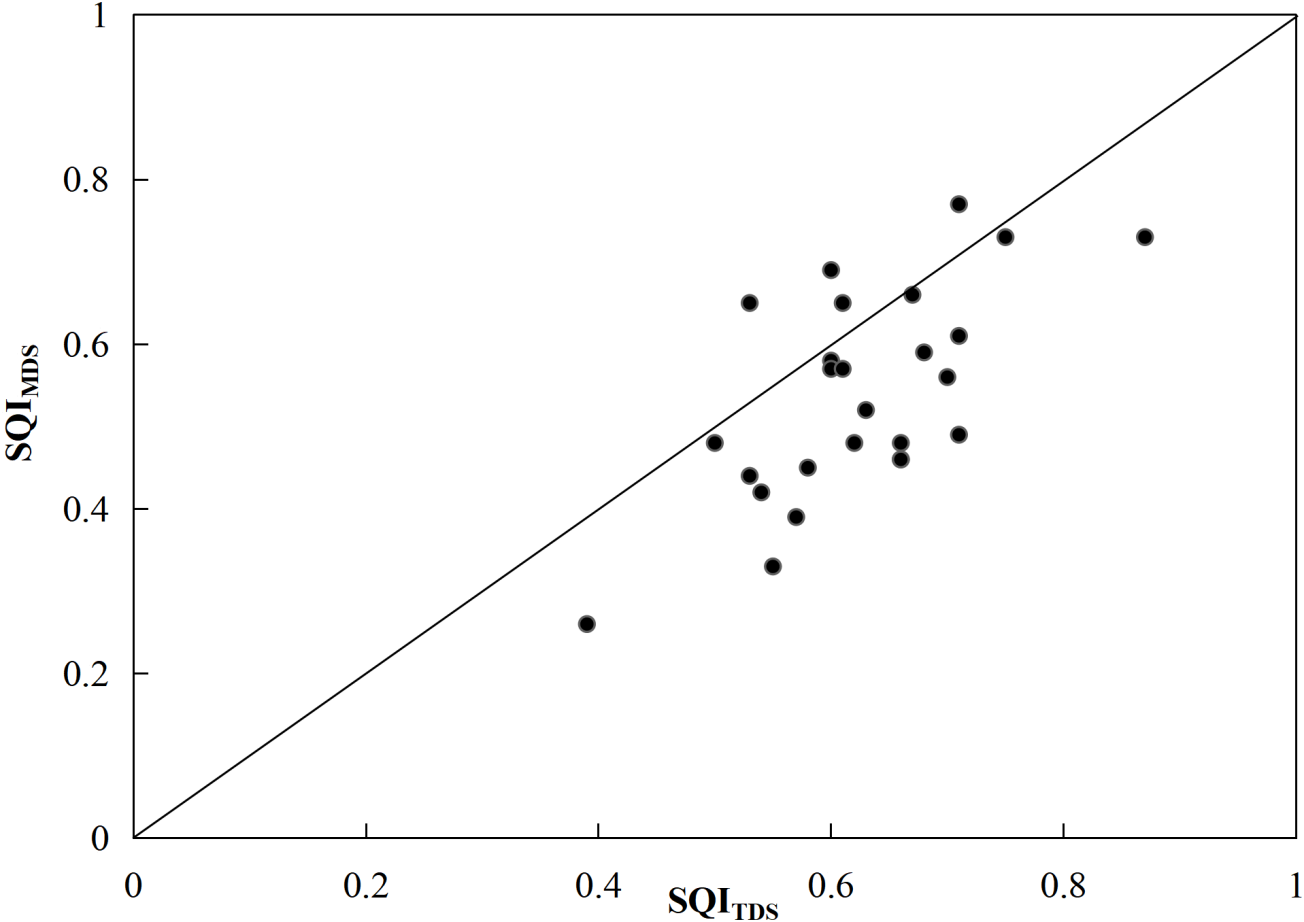
Comparison of the SQI_TDS_ and SQI_MDS_ results.

To determine the accuracy of the MDS method in evaluating soil quality in terms of artificial forest soil quality in the Qinling Mountains, the SQI_MDS_ and SQI_TDS_ results. The mean SQI_TDS_ value was 0.53 ± 0.013, with a coefficient of variation (CV) of 24.14%. The mean SQI_MDS_ value was 0.62 ± 0.09, with the coefficient of variation of 13.00%. The SQI_MDS_ range was relatively small (CV < 20%), and the SQI_TDS_ range was relatively large (CV > 20%). Obviously, the TDS method was more sensitive than the MDS method in reflecting the variation in the soil quality index. The difference between the mean SQI_TDS_ and SQI_TDS_ values was small, and the correlation between them was high (*R*^2^ = 0.702**, *n* = 26). The 1:1 line better reflects the consistency of the two comparison objects. As shown in figure, the SQI_TDS_ and SQI_TDS_ values were evenly distributed on both sides of the 1:1 line and were very close to the 1:1 line (Fig 7). The data points fluctuated relatively little on both sides of the 1:1 line, indicating that the SQI_TDS_ and SQI_TDS_ values revealed had a good corresponding relationship and that the MDS calculation and evaluation results were highly accurate. Furthermore, the coefficient of certainty (EF) and the coefficient of relative deviation (ER) [40] between the SQI_MDS_ and SQI_TDS_ values were calculated. The coefficient of certainty was 0.778 and the coefficient of relative deviation was 0.037, which indicated that the SQI values calculated by the BD, pH, SOM, SP, and SWC indicators in the MDS were close to the calculated values of all the indices. Thus, the MDS method is highly accurate and could be substituted for the TDS method for soil quality evaluation. The cost and time needed to evaluated the SQI in artificial forestland soil can be greatly reduced using the MDS method, based on a weighted approach using PCA and the norm value in the Qinling Mountains.

The indicators frequently incorporated into soil quality evaluation indices include the BD, pH, SOM content, TN content, TP content, AK content, and AP content [60]. The selection of soil quality evaluation indicators varies across different regions and research subjects. Mei L et al. [61] selected the BD, AP content, and AK content to construct the MDS. Liu KY et al. [62] chose the TN, TK, and AP contents to construct the MDS. Research conducted in the Qinling Mountains has also contributed to the methodology for MDS development. To establish the MDS, Zhang L [63] selected eight indicators, including the pH and organic matter, from a pool of 35. Zhang YJ [64] selected soil BD, pH, SOM content, and AP content, and Zhang CB [65] selected the SWC and SOM content to construct their respective MDS. The MDS indicators selected in this study show a certain degree of similarity with the aforementioned results, indicating that the minimum dataset evaluation index system in this research has good representativeness.

Among the indicators selected for the MDS, the BD is a common physical indicator for soil quality evaluation and is directly linked to soil structure, function, and ecological service capacity. The SWC serves as a pivotal factor driving the multifunctionality of soil quality by regulating physical structure, nutrient transport, and biological activity. In soil quality assessment, it is both a sensitive indicator and a background variable that explains the variation in other indicators. The soil pH indirectly affects plant nutrient uptake and utilization by regulating nutrient availability and microbial community structure. Owing to its comprehensive nature and simple and stable measurement, it is one of the most frequently included indicators in the MDS. The SOM and AP contents are essential nutrients for plant growth. In this study, the SOM content was significantly positively correlated with aggregate stability and available nutrient content, confirming its status as a core driver of soil quality. The AP content has become an indisputably representative indicator of phosphorus because of its high sensitivity, direct ecological function, and independent statistical contribution. The exclusion of the TN and TP contents from the MDS is primarily attributed to their relatively high background concentrations in the study area, which resulted in a weaker discriminatory power and lower explanatory contribution to soil quality variation compared with the other selected indicators. This selection highlights the function-oriented nature of soil quality evaluation. This study also demonstrated a correlation between the SQI derived from the TDS and that derived from the MDS. The MDS indicators can replace for the TDS indicators in measuring the soil quality of different forest stand types, which is consistent with the conclusions of previous studies.

## Conclusions

To evaluate the soil quality in the artificial forestland soil of the Qinling Mountains in China, 13 chemical and physical indicators were measured. The key soil quality indicators selected using correlation analysis, the norm value, and PCA were the BD, pH, SOM content, SP content and SWC content in the MDS. The SQI using that the soil quality level was middle, whereas the SQI level using the MDS method was higher. The spatial distribution characteristics of the SQI_TDS_ and SQI_MDS_ values were similar and showed a decreasing trend from the lower left to the upper right on the slope. Moreover, the SQI_MDS_ and SQI_TDS_ results were compared by the 1:1 line, and the coefficient of certainty and the coefficient of relative deviation indicated that the MDS method has high accuracy and could substitute the TDS method for soil quality evaluation in -the artifical forestland. Therefore, the MDS method and key soil quality indicators including the BD, pH, SOM content, SP content and SWC, can be applied to evaluate soil quality for artificial forestland in the Qinling Mountains. Furthermore, to address the challenges of high altitude, steep slopes, thin soil layers, and ecological fragility in the Qinling Mountains, a three-dimensional protection system based on “root–soil–vegetation” synergistic stabilization is constructed to improve soil quality in artificial forests. (1) **Direct Root Protection and Promotion Measures.** Local litter, branches, mosses, ferns and so forth should be applied to cover the ground around the tree roots. Organic fertilizer or imycorrhizal fungi should be applied within the main root distribution area. (2) **Water and Soil Conservation Engineering Measures on the Slope, such as Fish-Scale Pits or Hole-Based Land Preparation.** Semicircular pits should be excavated on the uphill side of trees, and the pit edges should be reinforced with stones or turf. Native shrubs and grasses, such as *Lespedeza bicolor*, and sea buckthorn, *can be planted to stabilize the root system.* (3) **Forest Management and Silvicultural Measures.** Large-scale monocultures should be avoided, and multilayered mixed forests of trees, shrubs, and grasses should be established. For instance, mixed forests of *Quercus* species and *Pinus armandii*, or mixed forests of *Tsuga chinensis* and *Pinus tabuliformis should be cultivated*.
