# Supplementary material for: Soil quality assessment based on the TDS and MDS methods in the Qinling mountains of China
Source: PLoS One. 2026 Aug 13;21(8):e0335384. doi: 10.1371/journal.pone.0335384 (PMC13472376; doi:10.1371/journal.pone.0335384)
Supplement: Supplementary file 2 [file pone.0335384.s003.docx]

**Review PONE-D-25-548225**

The manuscript entitled: “Soil Quality Assessment Based on the TDS and MDS Methods in the Qinling Mountains of China” is interesting and is in the scope of the journal. Authors conducted A comprehensive investigation on Soil Quality Assessment Based on the TDS and MDS Methods. Study was conducted in five artificial.

This article is well written, addresses an important topic, and provides data on the comparison of two methods of soil quality assessment. Although the results are clearly presented, we believe the authors have not discussed them. The reading of the results/discussion section needs improvement.

There are a number of issues with, abstract, introduction**,** results and discussion that need to be clarified/addressed. Below are more specific comments by section:

**Abstract:**

Lines 22, 26 and 28: replace “date” by “data” in the sentences

**Keywords:**

Lines 47: replace “date” by “data” in the sentence

**Introduction**

Lines 70: replace “date” by “data” in the sentences ‘Total data set’

**Laboratory analysis**

Line 152: add a space between reference and word

Lines 158-159: define TN (total nitrogen) and AP (available phosphorus)

**Soil quality assessment**

Lines 47: replace “date” by “data” in the sentence

**Results and discussion**

Line 285: Table 2 is not incorporate

**The spatial characteristic of soil physical and chemical indicators**

Lines 290-292: the authors indicated that ‘The soil BD increased from the down slope to the up slope. However, they reported also that ‘The maximum value BD was located in the middle of the slope and the minimum value of BD was located in the down slope’. Can they **write “The soil BD increased from the down slope to the middle of the slope”**

**Lines 299-301:** The spatial distribution of SOM was patchy and striped. The SOM was decreased from the down slope to the up slope. The maximum value of SOM was located in the middle and lower part of the slope and the minimum value of SOM was located in the top of the slope. **See my above comment**

**Conclusion:** Although the results are clearly presented, we believe the authors have not discussed them. The reading of the results/discussion section needs improvement.

I recommend that the discussion be thoroughly reviewed before the publication of this interesting article.
