# Supplementary material for: Soil quality assessment based on the TDS and MDS methods in the Qinling mountains of China
Source: PLoS One. 2026 Aug 13;21(8):e0335384. doi: 10.1371/journal.pone.0335384 (PMC13472376; doi:10.1371/journal.pone.0335384)
Supplement: Supplementary file 3 [file pone.0335384.s005.doc]

**Responses to Editor’s and Reviewers’ Comments**

**Manuscript ID:** PONE-D-25-548225

**Title:** Soil Quality Assessment Based on the TDS and MDS Methods in the Qinling Mountains of China

**Journal name:** PLOS ONE

Dear Editor and Reviewers,

Thank you very much for your careful review and constructive comments related to our manuscript. All suggestions and comments are helpful to improve the quality of our manuscript. We have studied all comments carefully and made a great effort to revise the manuscript accordingly. Once again we appreciate the reviewers’ efforts and time. If you have any question about this paper, please don’t hesitate to let us know.

The key actions taken are as follows:

i）The manuscript has been reformatted according to the provided style templates.

ii）Required statements regarding field permits and data availability have been added.

iii）All funding information has been removed from the manuscript text and correctly entered in the submission system.

iv）The copyrighted map in Figure 1 has been replaced with a new map created using public domain resources from.

v）We have ensured that all figures are properly cited within the text.

We hope that the revised manuscript is suitable for publication.

Revised sections were marked in red color in the revised manuscript. The point-by-point responses to the editor’s and reviewers’ comments/questions are detailed below.

Yours sincerely,

Weige Yang

23- Apr-2026

**Responses to the comments from Reviewers:**

Review Comments to the Author
Please use the space provided to explain your answers to the questions above. You may also include additional comments for the author, including concerns about dual publication, research ethics, or publication ethics. (Please upload your review as an attachment if it exceeds 20,000 characters)

**Response:** Thank you very much. We have explained the answers to the questions above.

**Reviewer #1:**

This literature evaluates the soil quality of artificial forests using two different SQIs. The overall structure is clear, but there are still some details that need to be improved. There are several questions and suggestions regarding this study.

**Response:** We have revised the manuscript according to your help suggestions. The following responses are presented in more detail.

**For the specific comments:**

1. The article mentioned that the study area belongs to a temperate semi humid monsoon mountain climate, but does not provide detailed information on the vegetation types, soil type distribution, and specific locations and areas of different artificial forests in the study area. Suggest supplementing this information to help readers better understand the research background and sample representativeness.

**Response:** Thank you for your valuable suggestion. We have supplemented detailed information on the research background in the revised manuscript.

Specifically, we have added climate, vegetation and soil in section of “Study Area” :

Climate: The study region belongs to the warm temperate semi-humid continental monsoon climate characterized by hot, rainy summers and mild, relatively dry winters.

Vegetation: The dominant vegetation types include deciduous broad-leaved forests and mixed deciduous-evergreen broad-leaved forests.

Soil: Luvisols (World Reference Base for Soil Resources) is the dominant soil type in the area.

Artificial Forests: We focused on the most widespread plantation types in the region, which are *Walnut*, *Robinia pseudoacaci*, Chinese pine (*Pinus psammopHila*), black locust (*Robinia pseudoacacia*), and secondary forests dominated by native oak species (*Quercus palustris Münchh*). These species represent the key silvicultural choices for soil and water conservation and timber production in the southern Qinling Mountains.

Please see the lines 107-120.

“The topographical features in this region is low mountain and hill, with higher elevations in the northwest and lower elevations in the southeast. Its landscape is typified by the structural ratio of 70% mountains, 20% cultivated land/terraces, and 10% rivers and water bodies**.** The study region belongs to the warm temperate semi-humid continental monsoon climate characterized by hot, rainy summers and mild, relatively dry winters. The mean annual temperature is 13.5 °C. The average annual precipitation is 758 mm and the 50% of the rain is received in the period from July to September. Luvisols (World Reference Base for Soil Resources) is the dominant soil type in the area. The dominant vegetation types include deciduous broad-leaved forests and mixed deciduous-evergreen broad-leaved forests. We focused on the most widespread plantation types in the region, which are *Walnut*, *Robinia pseudoacaci*, Chinese pine (*Pinus psammopHila*), black locust (*Robinia pseudoacacia*), and secondary forests dominated by native oak species (*Quercus palustris Münchh*). These species represent the key silvicultural choices for soil and water conservation and timber production in the southern Qinling Mountains. ”

A new Table 1 now provides key details for each plantation type, including location characteristics (elevation, aspect and slope) and stand age, enhancing sample representativeness transparency. Please see the lines 136.

**Table 1 Basic Characteristics of the soil sampling plots**

| **Forest type** | **Attitude (m)** | **Slope (°)** | **Aspect** | **Age（a）** |
| --- | --- | --- | --- | --- |
| *Walnut* | 760 | 0 | Southeast | 12 |
| *Robinia pseudoacaci* | 780 | 5 | Southeast | 21 |
| *Broussonetia papyrifera* | 800 | 40 | Southeast | 21 |
| *Pinus psammopHila* | 830 | 50 | Southeast | 21 |
| *Quercus palustris Münchh* | 860 | 60 | Southeast | 21 |

2. The article mentioned that a total of 26 soil samples were collected, but did not specify the distribution of the samples, such as whether they were evenly distributed under different terrain conditions such as altitude and slope. Suggest supplementing sample distribution information to enhance the persuasiveness of research results.

**Response:** Thank you for raising this important point regarding sample distribution. We agree that clarifying the spatial layout of our sampling design is crucial for interpreting the results. We have now supplemented detailed information in the revised manuscript.

Specifically, we have added the following to the section of “Soil Sampling”:

Sampling Strategy: Second, a grid of 50 m length × 20 m width was used for collecting soil samples to capture the full range of environmental gradients on the hillslope.

Sample Distribution Details: A suite of site characteristics, including including location characteristics (elevation, aspect and slope) and stand age for the samples（Table 1).

**Table 1 Basic Characteristics of the soil sampling plots**

| **Forest type** | **Attitude (m)** | **Slope (°)** | **Aspect** | **Age（a）** |
| --- | --- | --- | --- | --- |
| *Walnut* | 760 | 0 | Southeast | 12 |
| *Robinia pseudoacaci* | 780 | 5 | Southeast | 21 |
| *Broussonetia papyrifera* | 800 | 40 | Southeast | 21 |
| *Pinus psammopHila* | 830 | 50 | Southeast | 21 |
| *Quercus palustris Münchh* | 860 | 60 | Southeast | 21 |

Please see the lines 130-136.

3. The article mainly used TDS and MDS methods for soil quality assessment, but does not compare and analyze the advantages and disadvantages of these two methods in depth. Suggest adding relevant content.

**Response:** Thank you for this insightful suggestion. We agree that a deeper methodological discussion would strengthen the paper. We have now added a dedicated subsection comparing TDS and MDS in the revised manuscript.

Specifically, we have supplemented the following content in Intruduction.

Advantages and limitations: The advantage of the TDS method was that all measured indicators were analyzed and contained all soil information and the results are accurate and scientific. But the disadvantage was that the calculation requires a lot of work and the process was complicated. The strength of MDS method is **efficiency and parsimony** and its associated challenge is the **subjectivity and statistical uncertainty** inherent in the indicator selection process.

 Justification for our use: We clarify why employing both methods was valuable for our study: TDS provided a robust, full-information baseline, while MDS offered a pragmatic tool for identifying the most influential, manageable indicators for potential future monitoring. This dual approach enhances the credibility and utility of our assessment.

Please see lines 75-80 and 92-95..

4. Please explain why Pearson correlation analysis was chosen in data analysis and whether the normality of the data needs to be considered.

**Response:** Thank you for raising this important methodological point. We appreciate the opportunity to clarify our analytical choices.

We selected Pearson correlation analysis for the following key reasons: (1) to quantify the strength and direction of the relationships between the 13 soil properties. Because Pearson's *r* is the standard and most interpretable measure for assessing linear associations between the variables. (2) to identify the correlations between the selected indicators in the Minimum Data Set by Pearson’s r and to analyses the redundancy subsequently. (3) to compare our findings with a large body of existing literature on soil property relationships, enhancing the contextual relevance of our results.

The following text has been added to the data analysis section.

Please see lines 233-241.

“Pearson correlation analysis was employed to assess linear relationships among soil properties and between these properties and the Soil Quality Index (SQI). This method was chosen due to its appropriateness for continuous data and its prevalence in soil science, facilitating comparison with prior studies. Prior to analysis, the normality of variables was assessed using the Shapiro-Wilk test. Variables deviating significantly from normality were log-transformed to better meet the assumption of bivariate normality. The robustness of the linear correlation results was further verified by comparing them with non-parametric Spearman rank correlations, which yielded consistent patterns.”

5. The selection indicators and other content in lines 367-371 should belong to the Materials and Methods section;

**Response:** Thanks for your careful review and constructive suggestion. You are absolutely correct. The content in lines 367-371, which details the criteria and process for indicator selection, is foundational to the methodology and should be presented in the “Materials and Methods” section for better logical flow and readability.

We have moved this paragraph to the Section of “**Soil Quality Assessment**”, which is more appropriate based on the specific content. This adjustment ensures that readers encounter the methodological details in the correct sequence before reaching the “Results” section.

We appreciate your guidance in improving the manuscript’s structure.

Please see the lines 198-201.

“Furthermore, the indexes selected into the MDS must be uncorrelated with each other. The correlation analysis was carried out in each group. If *r* < 0.5, all indexes were selected into the MDS. If *r* > 0.5, the indicator, of which the Norm value was the largest, was selected into the MDS.”

6. The article mentioned that the spatial distribution of SQI is strip-shaped, but does not provide a detailed analysis of the reasons for this distribution. Suggest analyzing the formation mechanism of SQI spatial distribution based on factors such as terrain, soil type, vegetation type, etc

**Response:** Thank you for this insightful and constructive suggestion. We agree that delving into the underlying mechanisms behind the observed spatial pattern of the Soil Quality Index (SQI) would significantly enhance the depth and value of our discussion. we have significantly expanded the “**Soil quality evaluation using TDS**” section to to include a dedicated analysis of the potential drivers for the strip-shaped distribution of SQI.

Please see lines 364-379.

“It is evident that the soil quality level is closely related to slope position. This pattern of enrichment at lower slopes and depletion at ridge tops is a typical manifestation of spatial heterogeneity in soil properties within mountainous ecosystems. Studies have indicated that broadleaf forests are more effective in maintaining soil fertility and promoting soil nutrient functions [47]. However, this study found that the mean value of soil quality of *Broussonetia papyrifera* was lower than that of *Pinus psammopHila*. This may be attributed to the shallow root system and strong horizontal expansion of *Broussonetia papyrifera*, along with its fine root secretions rich in oxalic acid and citric acid. Concurrently, the decomposition of litter in coniferous forests generates higher levels of organic acids [48], which can activate aluminum- and iron oxide-bound phosphorus in the soil, leading to an accumulation of soil nutrients. Studies have shown that similar site conditions, different forest could lead to variations in soil nutrients and tree growth [49]. Although the natural conditions of the five forest in the study area are similar, the driving factors of soil quality (such as nutrients, microtopography, slope position, and so on.) differ among them. The direct or indirect interactions among these factors may influence soil quality.”

7. The article analyzed the results of soil quality assessment, but did not compare them with other research findings. Suggest adding relevant content, such as comparing soil quality with other areas or different types of artificial forests in the Qinling Mountains, to highlight the uniqueness and importance of the results of this study and increase the depth of discussion.

**Response:** Thank you for this excellent suggestion. We agree that situating our findings within the broader scholarly context through comparative analysis is crucial for highlighting their significance and advancing the discussion.

In response, we have expanded the results and discussion to include a new subsection ( 4.3. The accuracy test of the result the minimum data set) dedicated to comparing our soil quality assessment results with relevant studies. The added content focuses on two key comparisons:

(1) Comparison with Other Regions: We compare the index in MDS in our study with the index in MDS from other regions (Mei et al., 2021 and Liu et al., 2024) . It is demonstrated that the indicators selected in our MDS possess broad applicability in other regions.

(2) Comparison within the Qinling Mountains: More importantly, we compare our results for these specific plantation types with published data on soil quality under different vegetation types (poplar forest，pine forest, mixed forest, privet forest and vineyard) (Zhang et al, 2025) or different forest types (Quercus aliena var, acuteserrata forest, Pinus tabuliformis forest, Betula albosinensis forest, Picea wilsonii forest, Pinus armandi-Quercus aliena var, acuteserrata mixed forest，and Tsuga chinensis-Pinus armandi mixed forest)Zhang et al, 2024）and different land-use types (forest, grassland, orchards, vegetable gardens, cropland, green belts) (Zhang et al., 2026) within the Qinling Mountains. This intra-regional comparison directly addresses the uniqueness of our findings.

This addition strengthens the scholarly contribution of our work by moving from a standalone presentation of results to an engaged dialogue with existing research. We believe it significantly improves the depth and impact of the discussion.

Thank you again for this valuable guidance.

Please see lines 522-554.

“The indicators frequently incorporated into soil quality evaluation index include BD, pH, SOM, TN, TP, available potassium, and available phosphorus content [63]. The selection of soil quality evaluation indicators varies across different regions and research subjects. Mei Lan et al. [64] selected BD, available phosphorus, and available potassium content to construct the MDS. Liu Keyi et al.[65] chose TN, total potassium, and available phosphorus content to build the MDS. Research conducted in the Qinling Mountains has also contributed to the methodology for MDS development. Zhang Le [66] selected eight indicators, including pH and organic matter, from a pool of 35 to establish the MDS. Zhang Yanjun [67] selected soil bulk density, pH, organic matter, and available phosphorus, Zhang Chengbo [68] selected soil water content and organic matter to construct their respective MDS. The MDS indicators selected in this study show a certain degree of similarity with the aforementioned results, indicating that the minimum dataset evaluation index system in this research has good representativeness.

Among the indicators selected for the MDS, BD is a common physical indicator for soil quality evaluation, directly linked to soil structure, function, and ecological service capacity. SWC serves as a pivotal factor driving the multifunctionality of soil quality by regulating physical structure, nutrient transport, and biological activity. In soil quality assessment, it is both a sensitive indicator and a background variable explaining the variation of other indicators. Soil pH indirectly affects plant nutrient uptake and utilization by regulating nutrient availability and microbial community structure. Due to its comprehensive nature, simple and stable measurement, it is one of the most frequently included indicators in the MDS. SOM and AP are essential nutrients for plant growth. In this study, SOM showed a significant positive correlation with aggregate stability and available nutrient content, confirming its status as a core driver of soil quality. AP has become an indisputably representative indicator for the phosphorus dimension due to its high sensitivity, direct ecological function, and independent statistical contribution. The exclusion of TN and TP from the MDS is primarily attributed to their relatively high background concentrations in the study area, which resulted in a weaker discriminatory power and lower explanatory contribution to soil quality variation compared to other selected indicators. This selection highlights the function-oriented nature of soil quality evaluation. This study also demonstrates a correlation between the SQI derived from the TDS and that derived from the MDS. The MDS indicators can substitute for the TDS indicators in measuring the soil quality of different forest stand types, which is consistent with the conclusions of studies.”

References

[64] Mei N, Gu Y, Li DZ, Liang Y, Yuan JC, Liu JZ, Ren J, Cai HG.Soil quality evaluation in topsoil layer of black soil in Jilin Province based on minimum data set. Trans Chin Soc Agric Eng. 2021; 37(12): 91-98. https://doi:10.11975/j.issn.1002-6819.2021.12.011

[65]Liu KY, Yang J, Jiang SN, Gu HY. Evaluation of differences in soil quality of Populus simonii × P. nigra of different stand ages in typical black soil area based on a minimum data set. Acta Ecol Sinica. 2024; 44(9):3623-35. https://doi:10.20103/j.stxb.202307091478

[66]Zhang L, Li CY, Gao CY, Guo F, Li YF. Soil health assessment in the Qinling reclamation area based on minimum data set and risk index. J Soil and Water Conserv. 2025; 39(2): 348-356. https://doi:10.13870/j.cnki.stbcxb.2025.02.007

[67]Zhang CB. Analysis and study on soil quality characteristics of different land uses in the Northern foot of Qinling Mountains. Yanan Univ.2024.

[68]Zhang YJ，Hua SH，Guo R, Wei JT，Zhang WJ，Wang X，Yuan J．Analysis of soil health variation characteristics and driving factors in different forest types of the Qinling Mountains. Acta Ecol Sinica. 2026; 46(2): 945-958．https://doi:10.20103/j.stxb.202504271004

8. The article mentioned soil quality protection measures, but does not provide specific suggestions and implementation plans. It is suggested to propose protective measures for soil quality in different artificial forests based on research results, such as fertilization and adjustment of planting structure.

**Response:** Thank you for this constructive suggestion. we have significantly expanded the “**Conclusions**” section to provide protective measures for soil quality in different artificial forests.

Please see lines 568-581.

“Furthermore, to address the challenges of high altitude, steep slopes, thin soil layers, and ecological fragility in the Qinling mountainous, a three-dimensional protection system based on “root-soil-vegetation” synergistic stabilization is constructed to cultivate soil quality in artificial forests. (1) **Direct Root Protection and Promotion Measures.** Apply local litter, branches, mosses, ferns and so on to cover the ground around tree roots. Use organic fertilizer or inoculate with mycorrhizal fungi within the main root distribution area. (2) **Water and Soil Conservation Engineering Measures on the Slope Such as Fish-Scale Pits or Hole-Based Land Preparation.** Excavate semi-circular pits on the uphill side of trees and reinforce the pit edges with stones or turf. Plant the native shrubs and grasses to stabilize root system,such as Lespedeza bicolor, sea buckthorn, and so on. (3) **Forest Management and Silvicultural Measures.** Avoid large-scale monocultures and establish multi-layered mixed forests of trees, shrubs, and grasses. For instance, cultivating mixed forests of Quercus species and Pinus armandii, or mixed forests of Tsuga chinensis and Pinus tabuliformis.”

This addition enhances the practical utility and translational value of our research for forest managers and policymakers. Thank you for prompting us to strengthen this important aspect of the manuscript.

**Reviewer #2:**

1. The manuscript entitled: “Soil Quality Assessment Based on the TDS and MDS Methods in the Qinling Mountains of China” is interesting and is in the scope of the journal. Authors conducted A comprehensive investigation on Soil Quality Assessment Based on the TDS and MDS Methods. Study was conducted in five artificial.

This article is well written, addresses an important topic, and provides data on the comparison of two methods of soil quality assessment. Although the results are clearly presented, we believe the authors have not discussed them. The reading of the results/discussion section needs improvement.

There are a number of issues with, abstract, introduction**,** results and discussion that need to be clarified/addressed.

**Responses:** Thank you very much. We have revised the manuscript according to your help suggestions. The following responses are presented in more detail.

**Abstract:**

2. Lines 22, 26 and 28: replace “date” by “data” in the sentences.

**Responses:** Thanks so much for pointing this out. We have replaced “date” by “data” in the Lines 22, 26 and 28.

The revised sentences are: However, data on soil quality level at different artificial forest soil in Qingling Mountains of China is very little. The purposes of this research were to estimate the level and spatial distribution characteristic of soil quality applying the total data set (TDS) and minimum data set (MDS) methods and verify the accuracy of the result using the MDS methods at different artificial forest soil in Qingling Mountains of China.

Please see lines 24, 27.

**Keywords:**

3. Lines 47: replace “date” by “data” in the sentence

**Responses:** Thanks so much for pointing this out. We have replaced “date” by “data” in the Lines 47.

The revised keywords are: “minimum data set; total data set; soil quality; soil quality index; principal component analysis; Qinling Mountains”.

Please see line 49.

**Introduction:**

4. Lines 70: replace “date” by “data” in the sentences ‘Total data set’

**Responses:** Thanks so much for pointing this out. We have replaced “date” by “data” in the Lines 71.

The revised sentences is: “At present, SQI were usually calculated by the principle component analysis (PCA) method [13, 22, 23], linear and multiple regression analysis [11, 21], total data sets (TDS) [24, 25], minimum data sets (MDS) [13, 24, 22, 23]. ”

Please see the line 72.

**Laboratory analysis:**

5. Line 152: add a space between reference and word

**Responses:** Thanks so much for pointing this out. We have added a space between reference [36] and word.

The revised sentences is: “Soil aggregate stability was determined by the slow wet sieving method [36] and it was expressed as the mean weight diameter (MWD). The MWD was calculated as follows:”

Please see the line 167.

6. Lines 158-159: define TN (total nitrogen) and AP (available phosphorus).

**Responses:** Thanks so much for pointing this out. We revised it accordingly in the Laboratory analysis.

The revised sentences are: “One portion was passed through the 0.25 mm mesh to assay soil organic matter (SOM) and total nitrogen (TN, the sum of all organic and inorganic nitrogen forms). One portion was passed through the 1 mm mesh to assay nitrate nitrogen (NH_3_^-^-N), ammonium nitrogen (NH_4_^+^-N), total phosphorus (TP), available phosphorus (AP, the phosphate fraction extractable by 0.5 M NaHCO₃, representing plant-accessible P) and pH. ”

“Soil TP and AP was measured by extracting samples with the 0.5 M NaHCO_3_ and assaying P calorimetrically employing molybdate [34]. ”

Please see the lines 152-155 and 174.

**Soil quality assessment:**

7. Lines 47: replace “date” by “data” in the sentence

**Responses:** Thank you very much for pointing this out. We feel very sorry for making this mistake. We have made correction and would be careful to avoid such mistakes in our manuscript.

We have modified this description as “The data analysis steps of using the TDS method were as follows” in the line 179.

**Results and discussion**

8. Line 285: Table 2 is not incorporate

**Responses:**Thank you for this constructive comment. We agree that it is essential to fully integrate all tables into the manuscript narrative. We have now incorporated Table 3 into the revised manuscript as follows:

**Table 3.** Correlation (r) between measured indicators in the study catchment.

| **Soil**  **properties** | BD | SWC | MWD | GWD | SM | SP | pH | SOM | TN | NH_3_^-^-N | NH_4_^+^-N | TP | AP |
| --- | --- | --- | --- | --- | --- | --- | --- | --- | --- | --- | --- | --- | --- |
| BD | 1 |  |  |  |  |  |  |  |  |  |  |  |  |
| SWC | 0.015 | 1 |  |  |  |  |  |  |  |  |  |  |  |
| MWD | –0.174 | 0.190 | 1 |  |  |  |  |  |  |  |  |  |  |
| GWD | -0.118 | 0.238 | 0.993** | 1 |  |  |  |  |  |  |  |  |  |
| SM | -0.967** | -0.003 | 0.230 | 0.180 | 1 |  |  |  |  |  |  |  |  |
| SP | –0.224 | 0.110 | 0.163 | 0.138 | 0.191 | 1 |  |  |  |  |  |  |  |
| pH | 0.016 | -0.039 | -0.155 | -0.130 | 0.027 | -0.287 | 1 |  |  |  |  |  |  |
| SOM | –0.023 | -0.105 | 0.430* | 0.409* | 0.113 | -0.226 | 0.219 | 1 |  |  |  |  |  |
| TN | 0.065 | 0.454* | 0.479* | 0.516** | -0.072 | 0.246 | -0.322 | -0.300 | 1 |  |  |  |  |
| NH_3_^-^-N | –0.276 | 0.257 | 0.189 | 0.184 | 0.250 | 0.034 | -0.600** | -0.277 | 0.450* | 1 |  |  |  |
| NH_4_^+^-N | –0.075 | 0.251 | 0.712** | 0.729** | 0.143 | -0.135 | -0.288 | 0.318 | 0.582** | 0.298 | 1 |  |  |
| TP | 0.228 | -0.019 | 0.148 | 0.161 | -0.160 | -0.029 | -0.154 | 0.157 | 0.180 | 0.114 | 0.265 | 1 |  |
| AP | –0.191 | 0.352 | 0.559** | 0.581** | 0.202 | 0.021 | -0.145 | 0.262 | 0.573** | 0.417* | 0.753** | 0.163 | 1 |

* Indicates significance level at *P* < 0.05; ** Indicates significance level at *P* < 0.01.

Please see lines 309-310.

**The spatial characteristic of soil physical and chemical indicators**

9. Lines 290-292: the authors indicated that ‘The soil BD increased from the down slope to the up slope. However, they reported also that ‘The maximum value BD was located in the middle of the slope and the minimum value of BD was located in the down slope’. Can they write “The soil BD increased from the down slope to the middle of the slope”

**Responses:** We sincerely thank the reviewer for this meticulous and constructive comment, which has helped us identify and correct an imprecise description in our manuscript. The reviewer is absolutely correct in pointing out the logical inconsistency between these two statements.

“The spatial distribution of BD showed non-linear variation along the slope. The soil BD increased from the down slope to the middle slope, where the maximum value BD ( 1.50 g·cm⁻³) was located. Then, it decreases towards the upslope. The spatial distribution of BD was complex, which may be influenced by the integrated effects of topography, erosion, and moisture redistribution along the slope. ”

Please see lines 314-319.

10. **Lines 299-301:** The spatial distribution of SOM was patchy and striped. The SOM was decreased from the down slope to the up slope. The maximum value of SOM was located in the middle and lower part of the slope and the minimum value of SOM was located in the top of the slope. **See my above comment**

**Responses:** Thank you for this important observation. We agree that the description of the spatial pattern of soil organic matter (SOM) in the original text was contradictory and unclear. The statement “decreased from the down slope to the up slope” (indicating a monotonic trend) conflicts with the subsequent observation that the maximum value was found in the “middle and lower part,” which suggests a non-linear, hump-shaped distribution.

We have revised the text to accurately reflect the observed pattern based on our data analysis. The corrected description is as follows:

“The spatial distribution of SOM was complex and irregular along the slope. The maximum values of SOM were located in the middle and lower part of the slope and the minimum value of SOM was located in the top of the slope. There were some local patches with different SOM contents, indicating that factors other than the overall slope gradient, such as local differences in soil erosion, water retention, and vegetation cover, may also affect the SOM distribution. Some circular or oval - shaped areas with relatively high or low SOM content were observed, suggesting the existence of local micro - environmental factors that influence the accumulation or loss of SOM. ”

Please see lines 325-332.

**Conclusion:**

11. Although the results are clearly presented, we believe the authors have not discussed them. The reading of the results/discussion section needs improvement.

I recommend that the discussion be thoroughly reviewed before the publication of this interesting article.

**Responses:** We sincerely thank the reviewer for the positive feedback on the clarity of our results and for the crucial suggestion to strengthen the discussion. We have thoroughly revised the discussion section to address this point.

Please see lines 364-379, 522-554 and 568-581.

**Comments to the Author**

1. Is the manuscript technically sound, and do the data support the conclusions?

The manuscript must describe a technically sound piece of scientific research with data that supports the conclusions. Experiments must have been conducted rigorously, with appropriate controls, replication, and sample sizes. The conclusions must be drawn appropriately based on the data presented.

Reviewer #1: Yes

Reviewer #2: Yes

**Response:** Thank you very much for the valuable advice. We have carefully studied all comments and suggestion and made revised the manuscript accordingly. We hope that revision could meet your requirements.

2. Has the statistical analysis been performed appropriately and rigorously?
Reviewer #2: Yes
Reviewer #2: Yes

**Response:** Thank you very much.

3. Have the authors made all data underlying the findings in their manuscript fully available?
The [PLOS Data policy](http://www.plosone.org/static/policies.action#sharing) requires authors to make all data underlying the findings described in their manuscript fully available without restriction, with rare exception (please refer to the Data Availability Statement in the manuscript PDF file). The data should be provided as part of the manuscript or its supporting information, or deposited to a public repository. For example, in addition to summary statistics, the data points behind means, medians and variance measures should be available. If there are restrictions on publicly sharing data—e.g. participant privacy or use of data from a third party—those must be specified.

Reviewer #1: Yes

Reviewer #2: Yes

Response**:** Thank you very much.

4. Is the manuscript presented in an intelligible fashion and written in standard English?
PLOS ONE does not copy edit accepted manuscripts, so the language in submitted articles must be clear, correct, and unambiguous. Any typographical or grammatical errors should be corrected at revision, so please note any specific errors here.

Reviewer #1: Yes

Reviewer #2: Yes

**Response:** Thank you very much.

[NOTE: If reviewer comments were submitted as an attachment file, they will be attached to this email and accessible via the submission site. Please log into your account, locate the manuscript record, and check for the action link "View Attachments". If this link does not appear, there are no attachment files to be viewed.

**Response:** Thank you very much. Yes, I will do according your reminding.

While revising your submission, please upload your figure files to the Preflight Analysis and Conversion Engine (PACE) digital diagnostic tool, <http://pacev2.apexcovantage.com/>. PACE helps ensure that figures meet PLOS requirements. To use PACE, you must first register as a user. Registration is free. Then, login and navigate to the UPLOAD tab, where you will find detailed instructions on how to use the tool. If you encounter any issues or have any questions when using PACE, please email us At [figures@plos.org](mailto:figures@plos.org). Please note that Supporting Information files do not need this step.

**Response:** Thanks for your information.

In compliance with data protection regulations, please contact the publication office if you would like to have your personal information removed from the database.

**Response:** Thank you very much.

Thanks again for your good suggestions. We deeply appreciate your hard work for our manuscript, and we look forward to receive comments from you and the reviewers, again.

Kind regards.

Weige YANG
