# Supplementary material for: Soil quality assessment based on the TDS and MDS methods in the Qinling mountains of China
Source: PLoS One. 2026 Aug 13;21(8):e0335384. doi: 10.1371/journal.pone.0335384 (PMC13472376; doi:10.1371/journal.pone.0335384)
Supplement: Supplementary file 4 [file pone.0335384.s006.doc]

**Responses to Editor’s and Reviewers’ Comments**

**Manuscript ID:** PONE-D-25-548225

**Title:** Soil Quality Assessment Based on the TDS and MDS Methods in the Qinling Mountains of China

**Journal name:** PLOS ONE

Dear Editor and Reviewers,

Thank you very much for your careful review and constructive comments related to our manuscript. All suggestions and comments are helpful to improve the quality of our manuscript. We have studied all comments carefully and made a great effort to revise the manuscript accordingly. Once again we appreciate the reviewers’ efforts and time. If you have any question about this paper, please don’t hesitate to let us know. We hope that the revised manuscript is suitable for publication.

Revised sections were marked in red color in the revised manuscript. The point-by-point responses to the editor’s and reviewers’ comments/questions are detailed below.

Yours sincerely,

Weige Yang

29- Jun-2026

**Responses to the comments from Reviewers:**

Review Comments to the Author
Please use the space provided to explain your answers to the questions above. You may also include additional comments for the author, including concerns about dual publication, research ethics, or publication ethics. (Please upload your review as an attachment if it exceeds 20,000 characters)

**Response:** Thank you very much. We have explained the answers to the questions above.

**Reviewer #1:**

The manuscript still contains a number of minor language and formatting errors, including repeated words, incorrect grammar, and possible typographical errors. Examples include expressions such as “were were measured”, “the the correlation analysis”, and inconsistent use of SQI abbreviations. These issues do not necessarily affect the scientific conclusions, but they reduce the readability and professionalism of the manuscript.

**Response:** Thanks for your comments to our manuscript. According to your suggestion， AJE was used to edit the whole manuscript. We have revised the manuscript according the editors’, reviewer’ and AJE’ comments and suggestions and explained the answers to the questions above.

**Reviewer #2:**

You have taken into account the reviewers' recommendations, further improving the quality of your article.

**Response:** We are truly grateful to Reviewer #2 for the constructive comments in the previous round. Your suggestions were instrumental in strengthening the quality of our work. We are pleased that the revisions meet your expectations.

**Comments to the Author**

1. If the authors have adequately addressed your comments raised in a previous round of review and you feel that this manuscript is now acceptable for publication, you may indicate that here to bypass the “Comments to the Author” section, enter your conflict of interest statement in the “Confidential to Editor” section, and submit your "Accept" recommendation.

Reviewer #1: All comments have been addressed

Reviewer #2: All comments have been addressed

**Response:** Thank you very much.

2. Is the manuscript technically sound, and do the data support the conclusions?
The manuscript must describe a technically sound piece of scientific research with data that supports the conclusions. Experiments must have been conducted rigorously, with appropriate controls, replication, and sample sizes. The conclusions must be drawn appropriately based on the data presented.

Reviewer #1: Yes

Reviewer #2: Yes

**Response:** Thank you very much.

3. Has the statistical analysis been performed appropriately and rigorously?

Reviewer #1: Yes

Reviewer #2: Yes

Response**:** Thank you very much.

4. Have the authors made all data underlying the findings in their manuscript fully available?

The [PLOS Data policy](http://www.plosone.org/static/policies.action#sharing) requires authors to make all data underlying the findings described in their manuscript fully available without restriction, with rare exception (please refer to the Data Availability Statement in the manuscript PDF file). The data should be provided as part of the manuscript or its supporting information, or deposited to a public repository. For example, in addition to summary statistics, the data points behind means, medians and variance measures should be available. If there are restrictions on publicly sharing data—e.g. participant privacy or use of data from a third party—those must be specified.

Reviewer #1: Yes

Reviewer #2: Yes

**Response:** Thank you very much.

5. Is the manuscript presented in an intelligible fashion and written in standard English?
   PLOS ONE does not copyedit accepted manuscripts, so the language in submitted articles must be clear, correct, and unambiguous. Any typographical or grammatical errors should be corrected at revision, so please note any specific errors here.

Reviewer #1: Yes

Reviewer #2: Yes

**Response:** Thank you very much.

[NOTE: If reviewer comments were submitted as an attachment file, they will be attached to this email and accessible via the submission site. Please log into your account, locate the manuscript record, and check for the action link "View Attachments". If this link does not appear, there are no attachment files to be viewed.

**Response:** Thank you very much. Yes, I will do according your reminding.

 To ensure your figures meet our technical requirements, please review our figure guidelines: https://journals.plos.org/plosone/s/figures

You may also use PLOS’s free figure tool, NAAS, to help you prepare publication quality figures: https://journals.plos.org/plosone/s/figures#loc-tools-for-figure-preparation.

NAAS will assess whether your figures meet our technical requirements by comparing each figure against our figure specifications.

**Response:** Thanks for your information.

Thanks again for your good suggestions. We deeply appreciate your hard work for our manuscript, and we look forward to receive comments from you and the reviewers, again.

Kind regards.

Weige YANG
